# Role of Distinct Macrophage Populations in the Development of Heart Failure in Macrophage Activation Syndrome

**DOI:** 10.3390/ijms23052433

**Published:** 2022-02-23

**Authors:** Jakub Kuna, Zbigniew Żuber, Grzegorz Chmielewski, Leszek Gromadziński, Magdalena Krajewska-Włodarczyk

**Affiliations:** 1 Department of Rheumatology, School of Medicine, Collegium Medicum, University of Warmia and Mazury, 10-900 Olsztyn, Poland; gchmielewski.gc@gmail.com; 2 Department of Pediatrics, Faculty of Medicine and Health Sciences, Andrzej Frycz Modrzewski Kraków University, 30-705 Kraków, Poland; zbyszekzuber@interia.pl; 3 Department of Cardiology and Internal Medicine, School of Medicine, Collegium Medicum, University of Warmia and Mazury, 10-900 Olsztyn, Poland; leszek.gromadzinski@uwm.edu.pl

**Keywords:** macrophages, macrophage activation syndrome, heart failure, myocarditis

## Abstract

Macrophage activation syndrome (MAS) is one of the few entities in rheumatology with the potential to quickly cause multiple organ failure and loss of life, and as such, requires urgent clinical intervention. It has a broad symptomatology, depending on the organs it affects. One especially dangerous aspect of MAS’s course of illness is myocarditis leading to acute heart failure and possibly death. Research in recent years has proved that macrophages settled in different organs are not a homogenous group, with particular populations differing in both structure and function. Within the heart, we can determine two major groups, based on the presence of the C-C 2 chemokine receptor (CCR2): CCR2+ and CCR2−. There are a number of studies describing their function and the changes in the population makeup between normal conditions and different illnesses; however, to our knowledge, there has not been one touching on the matter of changes occurring in the populations of heart macrophages during MAS and their possible consequences. This review summarizes the most recent knowledge on heart macrophages, the influence of select cytokines (those particularly significant in the development of MAS) on their activity, and both the immediate and long-term consequences of changes in the makeup of specific macrophage populations—especially the loss of CCR2− cells that are responsible for regenerative processes, as well as the substitution of tissue macrophages by the highly proinflammatory CCR2+ macrophages originating from circulating monocytes. Understanding the significance of these processes may lead to new discoveries that could improve the therapeutic methods in the treatment of MAS.

## 1. Introduction

One of the most life-threatening states in rheumatology is macrophage activation syndrome (MAS)—a condition belonging to the secondary hemophagocytic lymphohistiocytosis (sHLH) group. It occurs predominantly in the course of systemic juvenile idiopathic arthritis (sJIA) and systemic lupus erythematosus (SLE). It may, however, also develop in the course of Still’s disease, rheumatoid arthritis, and systemic sclerosis. The syndrome’s main characteristics are hyperproliferation and hyperactivity of T lymphocytes (CD8+), NK cells, and macrophages exhibiting hemophagocytic properties. This leads to overproduction of chemokines and proinflammatory cytokines, as well as an uncontrolled systemic inflammatory response, which results in aggravated coagulating disorders and damage to multiple organs. The frequency of fully symptomatic MAS is estimated at ca. 10% in patients with active sJIA, and its subclinical form is estimated to have a frequency of up to 30% [1,2,3,4]; in patients with SLE, it is estimated to occur at a rate of at 0.9–9% [5,6,7]. The mortality rate oscillates in the 20–30% range, depending on the source [8,9,10], with heart failure being the main cause of death.

As the clinical features of MAS in rheumatological diseases are very similar to those in familial HLH (fHLH), it is most often diagnosed using the criteria established by the International Histiocyte Society [11] in 2004, focused on the general symptoms (prolonged fever), irregularities in the hematopoietic system (hemophagocytosis—most often appearing in bone marrow; hyperferritinaemia associated with the destruction of circulating erythrocytes; cytopenia in two or more cell lines; low or suppressed activity of NK cells; splenomegaly), hypertriglyceridemia, and hypofibrinogenemia. However, in clinical practice, those criteria proved not sensitive enough, especially in patients with sJIA, and thus, a new attempt was made to update them: Ravelli’s criteria, published in 2005, take into account hepatomegaly and an increased level of transaminases, symptoms in the central nervous system (headaches, convulsions, and coma), and increased risk of bleeding [12]. Criteria established by MAS Study Group in 2011 additionally place significance on assessing the ESR deceleration caused by hypofibrinogenemia [13]. Those last criteria, as well as those from 2004, include hemophagocytosis in the bone marrow: the subclinical form of MAS may be present in up to 30% of active sJIA cases, and invasion of the marrow is the earliest detectable occurrence of MAS. This may, however, pose some problems with e.g., the extracting of material not affected by the disease—in such cases, it may be helpful to check for the presence of the CD163 receptor, characteristic for phagocytic macrophages, by means of its specific antibodies [14].

Notably, the above criteria do not go into detail regarding the failure of different organs and systems (beside the liver). This is because of the disease’s extensive symptomatology and varied course of illness. However, its presence in the cardiovascular system is not an uncommon occurrence: according to Minoi et al. (2014), it constitutes even up to 25% of cases (its main form being pericarditis; other forms include arrhythmia, heart failure, and cardiomegaly) [15].

This article aims to summarize new research on the ontogenesis of macrophages, and their function and significance in the pathogenesis of heart failure in the course of MAS.

## 2. Historical and Contemporary Classification of Macrophages

First discovered in 1882, macrophages are one of the largest and most varied groups of white blood cells in the organism; they populate all organs and systems, where they adjust to local conditions and perform various functions. Their nomenclature is dependent on the organ they populate: they are known as the Kupffer cells in the liver, Langerhans cells in the skin, microglia in the brain, etc. Together with monocytes and dendritic cells, they form the mononuclear phagocyte system (MPS), first described in the 1970s [16,17]. At that time, it was assumed that the main sources of macrophages were circulating monocytes originating from the bone marrow, which migrated to the appropriate tissues prompted by chemotactical agents and then diversified into fully matured macrophages, continuously refilling their depleting reserve [18]. This model has, however, been undermined by research in the last decade: as it turns out, not only do particular organs have macrophages divided into discrete functional groups, part of which regenerate autonomously, but their population also takes place as early as during embryogenesis [19]. This mechanism will be described in detail further in the article.

Traditionally, macrophages are divided into subtypes—M1 (proinflammatory) and M2 (anti-inflammatory)—depending on their response to the activating agents. The main differentiating factor is the way an activated macrophage metabolizes arginine [20]: in the proinflammatory form, metabolism based on nitrous oxide synthase (iNOS) produces NO [21,22]; in the anti-inflammatory form, the expression of arginase triggers the production of L-ornitine—the precursor to the polyamines essential to the cell’s survival—as well as collagen to repair the damaged tissues [23]. The so-called “classic” activation into the M1 variant is initiated by TNF-α, INF-γ, and bacterial components such as lipopolysaccharide (LPS). In response to those agents, macrophages begin displaying phagocytic activity and produce large quantities of proinflammatory cytokines, including TNF-α, IL-6, IL-1β [24], IL-12, and iNOS; cell surface expression of CD68 also occurs [24,25]. “Alternative” activation, i.e., into the M2 variant, is initiated through stimulation of IL-4, IL-10, and IL-13. Macrophages activated this way display an anti-inflammatory phenotype through the production of IL-10 and growth agents (VEGF, TGF-β), accompanied by an increased cell surface expression of CD163—the scavenger receptor for the hemoglobin–haptoglobin complex—and a decreased capability to present antigens to T-lymphocytes [26,27,28,29]. Further research has proved this dichotomy a very simplified view of the matter; however, it still functions with certain modifications. Currently, it is assumed that the M1 and M2 subtypes are extremes, between which there is a spectrum of possible forms of activation; attempts have been made to systematize them by creating new subgroups, such as M2a, M2b, M2c, or Mox [30,31,32].

Another significant factor in the selective recruitment and activation of immune cells are chemokines. They are a group of low-molecular-weight proteins divided into four subfamilies based on the configuration of two NH_2_-terminal cysteic radicals: C, CC, CXC, and CX_3_C. In the accepted nomenclature, a chemokine molecule is labeled with the letter L (ligand), and its specific G protein-coupled receptor with the letter R [33]. As an example, the chemokines CCL1, CCL2, CXCL2, and CX_3_CL1 play a significant role in the pathogenesis of the coronary heart disease [34,35]; CCL2, CCL5, CCL7, and CXCL1 have a role in acute myocardial ischemia [36,37,38]; and CCL2, CCL5, CXCL9, and CXCL10 are significant in the response to an infection (examined on the model of Trypanosoma cruzi infection) [39,40].

## 3. Macrophages in the Heart

### 3.1. Origins of Different Macrophage Groups

Most of the research leading to the significant progress in differentiating the populations and functions of macrophages in the heart has been conducted on mouse models. Both in a healthy heart and in pathological conditions, the myocardium is an immunologically active organ. The heart of an adult mouse contains ca 10^3^ leukocytes per 1 mg of tissue [41], among which macrophages constitute the largest percentage, followed by dendritic cells, B and T-lymphocytes, and lymphoid cells [41,42,43,44,45].

Macrophages within tissues are not a uniform cellular group; populations of both different genetic suits and functions dictated by gene expression can be found within a single organ. The differences in their cell surface protein makeup allow the division into particular functional groups. For macrophages in the myocardium of a mouse, the affiliation with a given population is determined by the presence of the CCR2 chemokine receptors. Another line of division is the expression of class-II MHC molecules. Beside determining the macrophage’s function, the above characteristics also identify its origin [46,47,48,49,50].

Macrophages can be detected in the mouse as early as embryonic day 6.5–7.0 (E6.5–E7.0), originating from the yolk sac cells. Together with progenitor erythroblasts and megakaryocytes, they constitute the first wave populating the developing organs in the process called the primitive erythropoiesis [51,52,53]. The second wave, also originating from the yolk sac, occurs in E8.0–E8.5 and consists of erythro-myeloid progenitor cells, which migrate into the liver, where they divide into various cell lines, including the monocytic line [54,55]. In the period of E11.0–E17.5, the liver becomes the main originator of embryonic hematopoiesis; another line of tissue macrophages originates from monocytes produced within it. From E17.5 onward, this role is taken over by the bone marrow [55,56,57].

During the development of a mouse’s heart, it is possible to determine three discrete populations of tissue macrophages: those originating from the yolk sac, from monocytes developing in the embryonic period, and from mature monocytes [58,59,60,61].

The first population can be distinguished in the epicardium area in E11.5. These macrophages, originating from the yolk sac and characterized by a low expression of the cell surface chemokine receptor CC2 (CCR2), are referred to as CCR2− [49]. They also display a low expression of MHC-II molecules (MHC-II low). These cells play a large role in the process of coronary angiogenesis. In E14.5, a population of CCR2+ MHC-II low macrophages appears and connects with the endocardium; however, their function in the development of the myocardium is unknown [49].

Three populations of macrophages can be discerned in the heart of an adult mouse, bearing the phenotypes CCR2− MHC-II low and CCR2− MHC-II high (both of embryonic origin, both from the yolk sac and embryonic monocytes), and CCR2+ MHC-II high (originating from circulating mature monocytes), as well as a population of CCR2+ MHC-II low monocytes [48,59,60,61,62].

Important research conducted by Bajpai G. et al. [50] determined the composition of macrophages in the human heart: in biopsy samples of the myocardium obtained during transplants from patients with ischemic and dilated cardiomyopathy, populations of CCR2− and CCR2+ macrophages were discovered, along with CCR2+ monocytes. Based on their expression of HLA-DR (the human homologue of MHC-II), subpopulations of these cells were ultimately divided into three groups: CCR2+ HLA-DR low monocytes, along with CCR2− HLA-DR high and CCR2+ HLA-DR high macrophages. The only significant difference was the lack of a population corresponding to the CCR2− MHC-II low macrophages in the mouse [51]. Still, this research facilitated the extrapolation of results obtained from mice onto humans.

### 3.2. Location, Functions and Replenishment of Distinct Macrophage Groups

All populations of tissue macrophages adapt to the organ they occupy and participate not only in its immune functions, but also in maintaining tissue homeostasis [16,63,64]. Macrophages originating from circulating monocytes assume these functions upon settling within a given organ [65]. Typical common functions include phago- and micropinocytosis of dead cell fragments, and probing of the local microenvironment for foreign microorganisms [59,60,61,66]. Recent research has shown that both CCR2− and CCR2+ macrophages may take part in the conduction of electrical potential—numerous macrophage cells were discovered within the atrioventricular node, connecting with the cardiomyocytes via connexin gap junctions Cx43. Eliminating those macrophages or the deletion of the Cx43 connexin from the macrophages correlated with various levels of disruption in the node’s conductivity, along with the occurrence of atrioventricular blocks. More research is required to determine if those mechanisms play a role in the development of arrythmias in various pathological conditions such as myocarditis, heart failure, or myocardial infarction. The presence of macrophages in the atrioventricular node was previously observed in both mouse and human hearts [62].

Additionally, both types of MHC-II high macrophages have the ability to present antigens and stimulate the response of T-lymphocytes (to date, this function has only been observed during in vitro tests, but there is no research confirming it in vivo) [59,60].

CCR2− macrophages, especially during embryogenesis, are predominantly associated with the epicardium, where they direct the proper development and remodeling of the coronary vessels (most likely via IGF1 and IGF2) [49]. Embryos without CCR2− macrophages display significant abnormalities in the formation and maturation of coronary vessels [58].

Another function of this population of macrophages is their participation in maintaining tissue homeostasis—compared to CCR+ macrophages, they exhibit higher activity in the phagocytosis of damaged and fragmented cardiomyocytes [67].

One of the most important functions of the CCR2− macrophages seems to be the regulation of repair processes in response to damage to the myocardium. This is particularly apparent in research conducted on newborn mice, where damage to the myocardium did not cause deterioration in either the systolic or diastolic function of the left ventricle, nor any depletion of the microvascular structure. K.J. Lavine et al. proved that compared to the CCR2+ population, CCR2− macrophages produce decidedly smaller amounts of TNF-α and IL-β in response to either LPS or LPS+ATP [58].

The main factor differentiating CCR2+ macrophages from their CCR2− counterparts is their strong proinflammatory action upon activation—this is caused by the presence of genes enabling the synthesis of select cytokines, including IL-1, as well as neutrophilic chemokines facilitating the recruitment and migration of circulating monocytes into the tissue. CCR2+ macrophages are activated by the exposition of their TLR9 toll-like receptor to mitochondrial DNA, most likely from damaged myocardium cells [68].

To date, only the active state functions of this macrophage population has been researched; their role in the resting state is unknown [58].

In response to an inflammatory agent occurring in the heart, tissue macrophages emit a cytokine signal recruiting additional cells to assist in dealing with the threat. All macrophages originating from circulating monocytes belong to the CCR2+ phenotype and exhibit an even higher expression of proinflammatory genes than their counterparts among tissue macrophages [69]. This mechanism is universal and can be observed in most tissues, although there are cases where macrophages of monocytic origin do not begin to populate the tissue after the inflammatory process ends—they have no permanent population in the central nervous system [70], while in the heart, peritoneum, or liver, they undergo permanent integration [23,61,70,71,72].

This is the primary means of replenishing the population of CCR2+ macrophages in the heart. The CCR2− macrophage population level is maintained through multiplication within the organ, which is their only method of replenishment. This mechanism loses effectiveness during prolonged inflammation, which may lead to partial or even total depletion of the macrophage population. The vacated niche can then be resettled by the CCR2+ cells. This has been confirmed by tests on mice: in damaged hearts, the population of CCR2− macrophages was rapidly depleted and replaced with a CCR2+ population from a large influx of circulating monocytes, which in turn steered the response in the proinflammatory direction, causing further damage to the myocardium. Inhibiting the inflammatory signal triggering the recruitment of proinflammatory macrophages resulted in preserving the population of CCR2−, and thus decreasing the inflammatory response and retaining the angiogenetic function, which lessened the extent of the damage [48].

Migrant macrophages resettling the niches vacated by the depletion of tissue macrophages do not share their genetic makeup. They lack the genes that are essential in repair processes, such as TIMD4, LYVE1, or IGF1. TIMD4, the phosphatidylserine receptor, participates in the process of efferocytosis [73,74], the absence of which increases the extent of ischemia [75]. LYVE1 joins with the hyaluronate in the soft muscles, participating in the vascular homeostasis processes [76], while IGF1 directly promotes angiogenesis [49]. It is thus easy to understand why the shuffling of macrophage populations can produce such vastly different consequences of inflammation, especially in the late stages.

### 3.3. Macrophage Behavior in Response to Myocardial Damage

Research conducted on mouse models has given us detailed insight into the dynamics of the immune system’s response to myocardial ischemia. Within the first day from the incident, the area affected by ischemia contained mainly tissue macrophages, both of the CCR2− and CCR2+ populations. CCR2+ macrophages of monocytic origin began appearing within the second day, and by day 4, they outnumbered the other populations by a significant margin. Most likely, it is the proinflammatory population of CCR2+ that activates first in response to damage. In models where particular populations were not present before inducing an infarction, considerable differences were observed in the late effects of myocardial damage: echocardiography performed 28 days after the ischemic episode in mice without the CCR2+ macrophages displayed a smaller akinetic area, better systolic function, and smaller size of the left ventricle compared to those in specimens without the CCR2− group. However, these manipulations had no effect on the size of the original necrosis area [69].

The same research team created a model that showed the differences in the structure and function of the myocardium after a transplant: 28 days from the operation, hearts without the CCR2+ tissue macrophages exhibited only a slight degree of fibrosis, while a decreased influx of neutrophiles and lower cytokine expression compared to hearts without the CCR2− population were observed as early as within two days from the operation [69].

One of the main causes of acute heart failure requiring transplants is myocarditis—it was proven to be responsible for one in nine cases of all heart failures [77]. There are numerous factors contributing to myocarditis, the most frequent of which are infections—both viral (enterovirus, parvovirus B19, HCV, and adenoviruses) [78,79,80] and bacterial (8–17% cases of myocarditis are caused by Gram-positive bacteria)—as well as parasites (e.g., Trypanosoma cruzi). In most cases, the inflammation ends with the removal of the infectious agent; the proinflammatory phase transitions into the anti-inflammatory phase, and repair mechanisms are triggered, resulting in the creation of fibrous tissue. In some cases, persisting viral proteins or RNA can extend the inflammatory phase and result in extensive fibrosis, which may produce the symptoms of dilated cardiomyopathy [81]. Beside infections, myocarditis may be caused by allergic reaction to a specific drug or other chemical substance—the apoptosis of myocardium cells triggers the ejection of the so-called DAMPs (Damage-/Danger-Associated Molecular Patterns) into the extracellular space, which triggers a sterile inflammatory response by the immune system, which then develops into myocarditis [79].

Among the general population, the percentage of myocarditis caused by autoimmune mechanisms is relatively low, while epidemiological data on patients with SLE and sJIA show that the disease may affect the heart to a various extent in up to 25% of cases. Animal models were created to reproduce the patterns of autoimmune myocarditis, known as EAM (experimental autoimmune myocarditis) [82]. Unlike what can be observed in the response to an infection, which exhibits a certain balance of destructive and remedial processes, the EAM model is dominated by a pathological inflammatory response against autoantigens [83], which in all cases leads to chronic heart failure [58].

First attempts have been made to treat myocarditis by blocking the signal triggered by the CCL2, which recruits proinflammatory macrophages. Although early results seem promising [84], no drugs utilizing this route have been registered to date. It must be noted that this kind of therapy cannot be used in patients with an active infection as proinflammatory macrophages are essential for combating foreign microorganisms. Furthermore, although the CCL2 chemokine recruits cells of a decidedly proinflammatory profile, blocking its specific receptors paradoxically results in a shift of macrophage activation in the proinflammatory direction—research conducted by Deci M. et al. recorded a 200% increase in the M1 to M2 phenotype ratio compared to the control group [85].

### 3.4. Macrophages in Different Types of Heart Failure

We can distinguish three major types of heart failure, based on the left ventricular ejection fraction (LVEF) parameter: heart failure with a preserved ejection fraction (HFpEF), characterized by LVEF over 50%, heart failure with a reduced ejection fraction (HFrEF) with LVEF below 40%, and the intermediate heart failure with a mid-range ejection fraction (HFmrEF). These conditions differ in both their triggering factors and their development mechanisms [86,87,88]—particularly types of autoimmune response and the resulting restructuring of extracellular space [89].

HFpEF develops primarily as a result of extra-cardiac factors. The major risk factors include female sex, older age, kidney diseases, or constituents of metabolic syndrome (obesity, arterial hypertension, or type 2 diabetes). The combination of systemic inflammation, abnormalities in coronary microcirculation, and progressing fibrosis of the extracellular space results in the stiffening of the walls of the heart and the development of diastolic dysfunction [88]. Although the role of macrophages in the development of heart failure is not well understood, it has been proven that arterial hypertension and aging processes lead to an increased influx of monocytes originating from the marrow and the spleen, which increases the population of macrophages in the heart. In these processes the MHC-II high macrophage populations begin displaying the profibrotic phenotype and secreting IL-10, which indirectly activates myofibroblasts. The prolonged myofibroblast activity occurring in this mechanism leads to an excessive accumulation of collagen, which in turn aggravates the diastolic dysfunction [90,91].

HFrEF is primarily associated with damage to myocardial cells caused by myocardial infarction, myocarditis, or valvular heart disease. Inflammation, endothelial dysfunction, and the replacement of dead tissue with fibrous tissue result in systolic dysfunction [92,93]. It must be noted that the correct balance between the inflammation and repair phases may be a factor in preserving the proper function of the heart. A prolonged or inadequately suppressed inflammatory reaction may lead to abnormal reconstruction of the myocardium and impairment of its systolic function [94].

There are a number of studies on the pathophysiology of the coronary artery disease, including the role of macrophages in the creation of unstable atheromatous plaques [95]. It is, however, an important etiological factor of HFrEF, so it is worth noting that one of the causes may be a prolonged inflammatory phase in the vascular wall (sustained, e.g., by circulating proinflammatory cytokines [96,97]), which results in the recruitment of circulating leukocytes and persisting proinflammatory polarization of macrophages. The hindered transition into the “resolution” phase may lead to the creation of thin collagen caps that are susceptible to damage, and the occurrence of cardiovascular incidents [98,99,100,101,102,103].

Multiple studies have shown an association between specific inflammatory biomarkers and heart failure (both HFpEF and HFrEF). Beside the classic marker that is CRP (which is an independent prognostic factor for adverse events in patients with heart failure) or NT-proBNP [104,105,106], an increased concentration of TNF- α, ST2, IL-1, IL-6, IL-8, Galectin-3 (Gal-3), and growth differentiation factor 15 (GDF15) was also observed [107,108]. Additionally, a correlation was observed between the concentration of TNF- α, the level of NT-proBNP, and the NYHA heart failure class [105,109,110]. In patients with HFrEF, an increased concentration of cytokines, particularly TNF- α and IL-6, correlates not only with the aggravation of the illness, but also the risk of death [111]. In turn, a high concentration of proinflammatory cytokines from preceding co-morbidities can help predict the risk of HFpEF occurring in patients without heart failure [112,113], while a high concentration of Gal-3 turned out to be associated with poor outcomes in a group of patients with already-present HFpEF [114].

## 4. Role of Various Cytokines in Macrophage Activation Syndrome

The syndrome’s nomenclature places the activated macrophages at the center; however, it is not their pathological structure or function but the means of their activation that constitutes the cause of illness. The so-called “cytokine storm” is a rapidly escalating overproduction of cytokines and their ejection into the bloodstream. It is extremely important to define which particular agents are actually referred to by this dramatic-sounding term. The most noteworthy of those are the proinflammatory IL-2, INF-γ, M-CSF, IL-1, IL-6, IL-18, and TNF-α. It must, however, be noted that significant amounts of cytokine inhibitors are also present alongside them, e.g., soluble TNF receptors or IL-1R antagonists [8,115,116,117].

This cascading overproduction of cytokines is caused by malfunctioning cytotoxic T-lymphocytes CD38 and NK cells, which are unable to induce apoptosis in their target cancerous cells or those infected by viruses. The central agent in this mechanism is perforin—a cytolytic protein that creates pores in the membranes of the attacked cells, allowing the ingress of proteolytic enzymes. In healthy conditions, perforin is synthesized and stored in sacs together with granzyme B. The sacs are transported along the cytoskeleton’s actin fibers to the point of contact between a lymphocyte and a suspect cell, then the material is released into the synapse created in this way, introducing granzyme B into the cell and inducing apoptosis [1,118,119].

Each of these stages is genetically coded—the first gene responsible for this process is the PRF1, which directly codes perforin. It was the first gene detected during research into the etiology of the fHLH [120]. Further work led to the identification of other genes: UNC13D, STX11, STXBP2, LYST, RAB27A, and AP3B1 [1]. Mutations in either of them extend the contact between cells, resulting in an excessive production of the proinflammatory cytokines triggering the cytokine storm [119].

Mutations in the above genes were detected in ca 40% of patients with sHLH [121,122]. In the case of fHLH, the mutations are strong enough to cause fully symptomatic HLH [123,124], so it may be assumed that in the remaining cases, a triggering agent is required. A model was thus proposed, in which genetic predisposition compounded with protracted inflammation (as is the case in SLE or sJIA) and an infectious agent triggers a cytokine storm [125].

Other models are also being researched to explain the development of MAS. One promising avenue of research constituted triggering select MAS symptoms in mice with mutations in the UNC13D, which were subjected to extended stimulation of the TLR9 receptor. The results suggested that the receptor plays a significant role in the development of the disease [126,127,128].

The most important cytokines escalating the immunological cascades that constitute the cytokine storm are INF-γ, IL-1β, IL-6, IL-18, and TNF-α.

The INF-γ cytokine is produced by NK and T cells activated through interaction with the antigen-presenting cell (APC). It is the primary trigger for activating macrophages and polarizing them toward the proinflammatory M1 phenotype [30,129,130]. Active macrophages also begin producing cytokines, including TNF—another of them directly associated with activating macrophages into the M1 form. Additionally, TNF blocks the signals potentially steering the activation toward the M2 form, which further escalates the cellular proinflammatory response [131,132].

IL-1β is a cytokine produced mainly by activated monocytes and macrophages. It triggers the activation of endothelial cells and leukocytes, and increases the production of IL-6 [126,133,134,135,136]. Although the full extent of IL-1’s role in MAS is yet unknown, its increased level has been observed in episodes of acute sJIA—a rapid increase in its concentration correlates with the risk of MAS occurring in these patients [127,137,138,139,140].

IL-6, produced by active macrophages (although it is unclear whether these particular cells are its source in MAS [141]), also correlates with sJIA [142]. It is responsible for the early phase of an inflammatory response, and its heightened concentration is observed in the course of sepsis [115,143]. Although its role in MAS has not been fully researched either, prolonged exposition to its high concentrations in mouse models with simultaneous stimulation of the TLR produced acute inflammatory responses accompanied by the cytopenia and hyperferritinemia characteristic of MAS [144]. In vitro tests have also revealed that prolonged exposition of immune cells to high concentrations of IL-6 lowers the cytotoxicity of NK cells by reducing the expression of perforin genes and granzyme B [123].

IL-18 belongs to the IL-1 family and is found mainly in endothelial cells and circulating monocytes. Together with IL-1β, it stimulates the production of IL-6 in monocytes and macrophages [145,146]. Its concentration is extremely high in sJIA and MAS [147,148], while in sepsis, rheumatoid arthritis, and SLE, it is only moderately heightened [149,150,151,152], and thus, can be a useful marker of early MAS [153].

The two main agents activating macrophages into the M1 phenotype are INF-γ and TNF. Active macrophages are first found in the bone marrow and often precede a fully symptomatic MAS. The main method of detecting them is histochemical staining of the CD163 receptors in obtained samples. CD163 are present only on cells of monocyte-macrophage origin, and their increased expression is characteristic for activated macrophages of both the M1 and M2 phenotypes [154]. In fully symptomatic MAS, active macrophages have the ability to release these receptors—it was postulated that the concentration of soluble CD163 (sCD163) may correlate with active inflammation, and thus, its detection could be useful not only in diagnosing MAS, but also in assessing the severity of its course [155].

It was established that cytokines such as TNF-α, IL-1β, and IL-6 play an important role in the development of heart failure in several clinical scenarios, and some attempts were even made to regulate their effects on heart tissue, yet no conclusive treatment proposals were established [156].

## 5. Possible Models of Myocarditis Development during MAS

The possible circumstances for the activation of an inflammatory response include ischemia, infection, and autoimmune processes. Chronic inflammation, such as those occurring in active SLE and sJIA, manifests through increased concentrations of various proinflammatory cytokines, including IL-6, IL-18, or INF-γ, which are known to trigger proinflammatory responses in macrophages. Prolonged inflammation compounded with a genetic defect in the coding of perforin creates a unique and extremely adverse environment, in which an infection that would otherwise be negligible could potentially trigger a snowballing cytokine reaction (“cytokine storm”) and the development of a fully symptomatic MAS.

There is currently no known research into the composition of macrophage populations in the heart during a cytokine storm or in the moments before its onset. It can be assumed that one of the three following scenarios may occur:

1. The heart has not yet been affected by the inflammation and possesses tissue populations of both CCR2− and CCR2+ macrophages. If the CCR2+ subtype is activated by the inflammation, the recruitment of monocytes, depletion of CCR2− and further development of the inflammation may occur with some delay.
2. The heart has no CCR2− macrophage population or their number is significantly diminished by prior inflammatory incidents. In this case, the activation of macrophages may prompt a strong and rapid inflammatory response.
3. Ongoing infection within the heart, e.g., endocarditis. The inflammatory process may be progressing in the heart even before the onset of a fully symptomatic MAS, in which case, a severe aggravation of the inflammatory response and its extent may occur.

**Figure 1:**
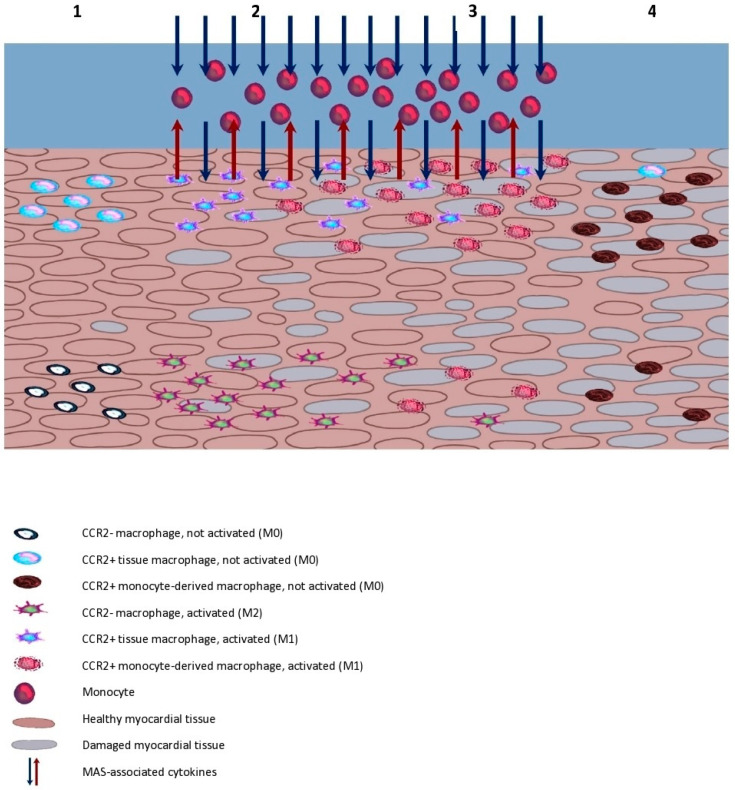
Changes of macrophage populations in myocardium during macrophage activation syndrome. 1. In a healthy myocardium, both groups of tissue macrophages, CCR2− and CCR2+, are present in their not-active state. 2. Tissue macrophages are being activated by cytokines associated with MAS, and by initial damage of heart tissue. They start producing their own cytokines and begin the recruitment of circulating monocytes. 3. Tissue macrophage populations are being depleted by prolonged inflammatory state and are replaced by monocyte-derived macrophages. Those newly recruited macrophages also produce cytokines and contribute to further damage of the myocardium. 4. After MAS is resolved, most of the original groups of macrophages are replaced by the now not-active monocyte-derived CCR2+ population. Moreover, tissue damage that occurred during the inflammation is likely to contribute to development of chronic heart failure.

In all of the above cases, even if the macrophage populations are intact at the first stage of the illness, time clearly works against the myocardium [Figure 1]. Even if MAS is contained and the heart was affected by the inflammation over its course, the long-term consequences of changes in the makeup of the phagocytic system may increase the risk of cardiovascular incidents and hasten the development of heart failure—this is already visible in patients with SLE, even without MAS incidents [157]. There are no current studies distinguishing the types of heart failure in the course of MAS. One analysis describing 103 MAS episodes in 89 patients with SLE indicates the presence of myocarditis in 21.4% of cases and pericarditis in 23.3% [158]. Although we lack precise data, it can be surmised that if a patient with MAS manifests the presence of myocarditis, it soon may be followed by rapidly developing HFrEF. It must, however, be taken into account that MAS occurs predominantly in patients with systemic autoimmune diseases, where a prolonged inflammation increases the risk of heart failure, cardiovascular disease, and myocardial infarction [159,160,161,162]. MAS occurring in this group may have the characteristics of myocarditis overlapping with already existing symptoms of heart failure.

## 6. Conclusions

Within the last two decades, it has been proven that tissue macrophages are a group of diverse cellular populations, each of which carries their own specific functions in different organs. In the heart, the CCR2− cells appear to be important participants and mediators in repair processes. Unfortunately, this cell group is the most prone to depletion over the course of inflammation. At the same time, the proinflammatory CCR2+ macrophages recruit other cells through the production of cytokines—mainly monocytes, which then transform into CCR2+ macrophages, exhibiting even stronger proinflammatory traits. In macrophage activation syndrome, this feedback loop leads to prolonged inflammation and may play a role in the development of potentially fatal acute heart failure. More research is certainly needed to understand of the processes leading to the very high mortality rate in MAS with heart failure being the main cause of death.
